# Stressful Life Events Experienced by Women in the Year Before Their Infants’ Births — United States, 2000–2010

**Published:** 2015-03-13

**Authors:** Elizabeth R. Burns, Sherry L. Farr, Penelope P. Howards

**Affiliations:** 1 Rollins School of Public Health, Emory University, Atlanta, GA; 2 Division of Reproductive Health, National Center for Chronic Disease Prevention and Health Promotion, CDC

Epidemiologic studies suggest that prenatal stress is associated with preterm birth, low birth weight (1–3), and peripartum anxiety and depressive symptoms (4). The most recent population-based study, assessing trends in stress experienced in the year before an infant’s birth, used 1990–1995 data from 11 states participating in the Pregnancy Risk Assessment Monitoring System (PRAMS) (5). That study found that 64% of women surveyed reported experiencing at least one stressful life event (SLE), although the prevalence declined slightly over the study period. PRAMS data for 2000–2010 were used to examine more recent trends and to determine if the prevalence of SLEs has continued to decrease in the past decade. Additionally, 2010 data were used to determine the extent that maternal demographics and state of residence are associated with SLEs. This report describes the results of those analyses, which found that most women in the sample reported experiencing ≥1 SLEs in the year before their infant’s birth, although the prevalence of experiencing SLEs decreased during 2000–2010. In 2010, based on data from 27 states, 70.2% of women reported ≥1 SLEs. The mean number of SLEs was 1.81, ranging from 1.41 in New York City to 2.26 in Oklahoma. SLEs were most frequently financial. Prevalence of SLEs varied by PRAMS reporting site and maternal demographics. Younger, less educated, unmarried, and Medicaid-covered women had the highest prevalence of SLEs. Public health practitioners and clinicians can use the information on trends and risk factors for SLEs to determine the likelihood that pregnant women might benefit from screening for stressors during pregnancy.

PRAMS is a population-based surveillance system administered by CDC in conjunction with state and New York City health departments ( http://www.cdc.gov/PRAMS ). PRAMS collects self-reported information on maternal experiences and behaviors before, during, and after pregnancy among women who delivered a live infant. Collection occurs annually, and, as of 2013, a total of 40 states and New York City participate, representing approximately 78% of all U.S. live births. Each site surveys by mail a stratified, systematic sample of 1,300–3,400 women identified from birth certificate data 2–6 months after a live birth. Up to three attempts are made to contact the woman by unregistered mail, followed by a maximum of 15 telephone calls per available telephone number to reach nonresponders. The PRAMS protocol was approved by institutional review boards at each site and CDC. Each participant provides written informed consent. Response rates must exceed 65% for the data from a site to be reported; 2010 response rates for the sites included in this analysis ranged from 65% to 83%.

PRAMS includes 13 questions about maternal SLEs experienced in the year preceding the birth of the child. Based on previous research (6), SLEs were grouped into four dichotomous constructs: 1) emotional stressors (family member was ill and hospitalized or someone very close died); 2) financial stressors (moved to a new address, lost job, partner lost job, or unable to pay bills); 3) partner-associated stressors (separated/divorced, argued more than usual with partner/husband, or husband/partner said he did not want pregnancy); and 4) traumatic stressors (homeless, involved in a physical fight, partner or self went to jail, or someone very close had a problem with drinking or drugs). Women who reported ≥1 SLEs in a group were categorized as experiencing the construct. Trends in prevalence of SLEs and the SLE constructs from 2000–2010 were assessed using five logistic regression models, one for each construct and one for overall SLEs. Infant birth year was used as the independent variable. Unadjusted trends and trends adjusted for maternal demographic characteristics (marital status, race/ethnicity, age, education, and Medicaid coverage during pregnancy and/or delivery) were examined. For statistically significant trends (p<0.05), annual percentage point change was assessed using logistic regression and estimated from the beta coefficient of the infant’s birth year. Using 2010 data only, prevalence of each construct, prevalence of experiencing ≥1 SLEs, and mean number of SLEs were calculated by PRAMS reporting site and by maternal demographic characteristics. Differences in prevalence of SLEs and mean number of SLEs by maternal demographic characteristics were assessed using chi-square tests and analysis of variance, respectively.

For trend analyses, data from 10 sites that participated in PRAMS every year during 2000–2010 and had sufficient response rates (≥65%) for all years* (n = 187,390 women) were analyzed. Prevalences by reporting site and maternal demographics for 2010 were estimated using data from 27 sites† (n = 38,255 women) that met or exceeded the response rate threshold. Women were excluded if they had missing data for one or more questions on SLEs (n = 6,488 for 2000–2010 [3.5%]; n = 1,364 for 2010 [3.6%]). All analyses were weighted to produce population-based estimates.

Modest but statistically significant decreases occurred during 2000–2010 in self-reported prevalence of ≥1 SLEs and all four constructs of SLEs (financial, emotional, partner-related, and traumatic) (p-value for trend <0.05 for all) (Figure). During 2000–2010 prevalence of ≥1 SLEs decreased 0.54 percentage points per year, financial stressors decreased 0.44 percentage points per year, emotional stressors decreased 0.35 percentage points per year, partner-related stressors decreased 0.58 percentage points per year, and traumatic stressors decreased 0.26 percentage points per year. Results remained significant after adjusting for maternal demographics. For all years, financial SLEs were the most frequently reported type of SLE, and traumatic SLEs were the least frequently reported type.

In 2010, the prevalence of individual SLE constructs, ≥1 SLEs, and mean number of SLEs varied by site (Table 1). For all sites combined, 51.0% of women reported ≥1 financial SLEs in the year before their infant’s birth (range = 42.2% in Georgia to 58.1% in Oklahoma), 29.6% reported ≥1 emotional SLEs (range = 22.3% in Georgia to 40.0% in West Virginia), 28.5% reported ≥1 partner-related SLEs (range = 22.7% in Utah to 35.5% in Arkansas), and 17.6% reported ≥1 traumatic SLEs (range = 11.3% in New Jersey to 25.9% in West Virginia). Overall, 70.2% of women reported ≥1 SLEs in 2010 (range = 58.5% in Georgia to 77.5% in West Virginia). In 2010, the mean number of SLEs was 1.81 (standard error [SE] = 0.02) overall and ranged from 1.41 (SE = 0.05) in New York City to 2.26 (SE = 0.09) in Oklahoma.

In 2010, the prevalence of experiencing SLEs in the year before the infant’s birth varied by the women’s demographic characteristics (Table 2). Women who were married, were aged ≥30 years, had ≥16 years of education, or had private insurance reported the lowest prevalence of all SLE constructs and reported the lowest mean number of SLEs. Prevalence of all constructs decreased with increasing age. Asian/Pacific Islanders reported the lowest point prevalence for all SLE constructs, and 95% confidence intervals did not overlap with any other racial/ethnic groups. Black women reported the highest point prevalence of emotional, partner-related, and traumatic SLEs; however, the 95% confidence intervals overlapped with other racial/ethnic groups. Unmarried women had the highest absolute mean number of SLEs (2.48; SE = 0.04), and Asian/Pacific Islanders reported the lowest mean number of SLEs (1.11; SE = 0.04).

## Discussion

The prevalence of the four SLE constructs during the year preceding a live birth decreased slightly during 2000–2010, and the downward trend remained statistically significant after adjusting for women’s demographic characteristics. In 2010, report of SLEs varied by site and demographic characteristics, with women in Oklahoma and West Virginia, younger women, less educated women, unmarried women, and women covered by Medicaid reporting the highest number of SLEs. However, more than 70% of women delivering a live birth in 2010 reported experiencing ≥1 SLEs, with financial SLEs the most commonly reported. A 2005 U.S. population-based survey reported that 40.1% of women in the general population experienced an SLE in the past year (7). However, these results are not directly comparable because of differences in methodology, stressors assessed, and survey year.

A previous study indicated that experiencing SLEs was common during 1990–1995, with 64% of women reporting ≥1 SLEs in the year before their infant’s birth (5). When the current analysis using 2010 data was restricted to the same SLEs included in the previous report, 62.9% (95% confidence interval = 62.6%–63.3%) of the sample reported experiencing ≥1 SLEs, consistent with the prevalence estimate for 1990–1995. The older study also reported that the prevalence of experiencing ≥1 SLEs varied by maternal demographics, with low socioeconomic status most strongly associated with experiencing an SLE (5). Similarly, 78% of women covered by Medicaid for prenatal care or delivery reported ≥1 SLEs in 2010.

The findings in this report are subject to at least five limitations. First, data were available for only 10 sites for 2000–2010, and only 27 sites for 2010 prevalence estimates; hence, generalizability might be limited. Second, PRAMS measures 13 SLEs during the 12 months before a live birth and not the perceived level of stress experienced by the individual woman nor whether the SLE occurred during pregnancy. Third, PRAMS relies on self-reported, retrospective data, and respondents might not accurately recall or report certain SLEs. Fourth, 3.6% of women with missing information on SLEs were excluded, which could underestimate the prevalence. Finally, nonresponse bias is possible because response rates ranged from 65% to 83%.

Current research suggests that increased prenatal stress is associated with adverse pregnancy outcomes, including low birth weight, preterm birth (1,2,3), and peripartum depression (4). However, there is evidence that social support has a mitigating effect on the relationship between stress and adverse pregnancy outcomes (1). Therefore, public health efforts to identify and reduce stress among pregnant women might benefit the psychological and physical health of pregnant women and their infants. To this end, in 2006, the American College of Obstetricians and Gynecologists published a committee opinion recommending that all pregnant women, regardless of socioeconomic status, education level, or race/ethnicity, receive psychosocial screening and referral, as needed, during their prenatal visits (8). Additionally, current American College of Obstetricians and Gynecologists antepartum care guidelines recommend that women be screened for psychosocial complications and social support (9).

Despite recommendations for screening, there is limited information on effective interventions for stress reduction in pregnant women. Approaches to reducing stress have primarily examined three avenues: 1) reducing physical stress through meditation or yoga; 2) increasing education; and 3) providing additional social support (1). The effectiveness of such interventions remains uncertain, but interventions such as group prenatal care for women at higher risk for SLEs have shown promise in increasing self-efficacy and satisfaction with care, which can contribute to increased psychosocial well-being (10). Clinicians should be aware that although SLEs are especially prevalent among low-income, younger, unmarried, and less educated women, most women with ≥16 years of education (59.6%), with private insurance (64.2%), and who are married (64.2%) also experience SLEs.

What is already known on this topic?

Current research suggests that stress experienced during pregnancy increases the risk for preterm birth and low birth weight. Current American College of Obstetricians and Gynecologists antepartum care guidelines recommend that women be screened for psychosocial complications and social support during their prenatal visits. Population-based estimates from 1990–1995 indicated that 64% of women experienced stress in the year preceding the birth of a live infant.

What is added by this report?

The prevalence of self-reported stressful life events (SLEs) decreased modestly but significantly during 2000–2010. Despite this, 70.2% of women reported ≥1 SLEs in 2010. The mean number of SLEs was 1.81, ranging from 1.41 in New York City to 2.26 in Oklahoma. SLEs were most frequently financial. Prevalence of SLEs vary by state and maternal demographic characteristics and are especially prevalent among younger women, women with <16 years of education, unmarried women, and women that were covered by Medicaid for prenatal care or delivery of their child.

What are the implications for public health practice?

These findings provide support to the recommendation by the American College of Obstetricians and Gynecologists that clinicians screen all prenatal care patients for psychosocial issues. Prenatal care clinicians should be aware of the prevalence of stress in their patients’ lives and provide referral to help alleviate stress, when needed.

## Figures and Tables

**FIGURE:**
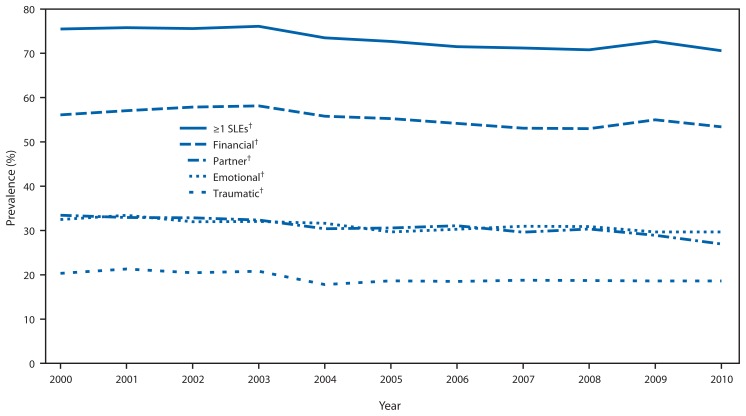
Prevalence of self-reported stressful life events (SLEs) in the year before an infant’s birth among mothers who had live births — Pregnancy Risk Assessment Monitoring System, 10 states,* 2000–2010 * Alaska, Arkansas, Colorado, Hawaii, Maine, Nebraska, Oklahoma, Utah, Washington, and West Virginia. ^†^ P-value for trend <0.05.

**TABLE 1:** Self-reported prevalence of stressful life events (SLEs) in the year before an infant’s birth among mothers who had live births, by site — Pregnancy Risk Assessment Monitoring System, 27 sites,* 2010

	≥1 financial SLE		≥1 emotional SLE		≥1 traumatic SLE		≥1 partner SLE		≥1 SLE total		Mean no. of SLEs	
						
State	%†	(95% CI)	%	(95% CI)	%	(95% CI)	%	(95% CI)	%	(95% CI)	Mean	(SE)
Alaska	49.6	(45.9–53.3)	26.5	(23.4–29.8)	21.0	(18.2–24.2)	26.6	(23.5–30.1)	68.7	(65.1–72.0)	1.73	(0.07)
Arkansas	57.1	(53.7–60.5)	35.9	(32.7–39.2)	25.9	(23.0–29.1)	34.5	(31.4–37.8)	78.7	(75.7–81.4)	2.22	(0.07)
Colorado	53.3	(50.2–56.4)	29.5	(25.8–31.4)	16.4	(14.2–18.8)	25.3	(22.7–28.1)	70.6	(67.6–73.3)	1.74	(0.06)
Delaware	49.9	(46.8–52.9)	31.7	(29.0–34.6)	18.1	(15.8–20.5)	29.0	(26.3–31.9)	71.4	(68.6–74.1)	1.83	(0.06)
Georgia	42.2	(37.4–47.1)	22.3	(18.5–26.6)	14.1	(11.0–17.9)	24.0	(20.0–28.4)	57.5	(52.6–62.3)	1.55	(0.11)
Hawaii	49.1	(45.9–52.3)	24.9	(22.3–27.8)	13.4	(11.4–15.7)	27.2	(24.4–30.1)	64.4	(61.2–67.4)	1.56	(0.06)
Maine	56.5	(53.0–60.0)	34.0	(30.7–37.5)	21.3	(18.4–24.5)	27.3	(24.2–30.7)	74.5	(71.4–77.4)	2.05	(0.07)
Maryland	50.4	(45.4–54.4)	28.9	(25.4–32.7)	16.0	(13.2–19.3)	27.8	(24.3–31.6)	69.3	(65.5–72.8)	1.80	(0.08)
Massachusetts	50.5	(46.9–54.1)	30.8	(27.6–34.3)	16.1	(13.6–19.0)	26.6	(23.6–29.8)	70.5	(67.2–73.7)	1.73	(0.07)
Michigan	53.0	(50.0–56.1)	34.1	(31.3–37.1)	19.2	(16.9–21.8)	31.4	(28.7–34.3)	73.8	(71.0–76.4)	1.92	(0.06)
Minnesota	47.4	(44.5–50.4)	26.6	(24.1–29.3)	15.4	(13.4–17.7)	25.0	(22.5–27.7)	65.4	(62.5–68.2)	1.55	(0.05)
Missouri	57.0	(53.9–60.0)	33.0	(30.2–36.0)	20.2	(17.7–22.8)	31.9	(29.0–34.9)	74.6	(71.9–77.1)	2.07	(0.07)
Nebraska	50.0	(47.3–52.8)	26.4	(24.0–29.0)	15.1	(13.3–17.2)	25.3	(23.0–27.8)	68.2	(65.6–70.7)	1.64	(0.05)
New Jersey	48.5	(45.7–51.4)	29.8	(27.2–32.4)	11.3	(9.7–13.2)	26.7	(24.3–29.2)	68.3	(65.6–70.9)	1.62	(0.05)
New York State§	50.6	(46.7–54.5)	30.7	(27.2–34.4)	18.0	(15.1–21.4)	27.9	(24.4–31.6)	70.0	(66.3–73.4)	1.76	(0.08)
New York City	43.1	(39.9–46.5)	23.9	(21.2–26.9)	13.2	(11.0–15.7)	25.9	(23.0–29.0)	64.8	(61.6–67.9)	1.41	(0.05)
Ohio	52.0	(48.3–55.7)	35.9	(32.4–39.5)	21.0	(18.2–24.2)	31.7	(28.4–35.2)	73.7	(70.4–76.8)	2.11	(0.08)
Oklahoma	58.1	(54.3–61.7)	33.4	(29.9–37.0)	24.3	(21.2–27.9)	32.9	(29.4–36.6)	74.3	(70.9–77.5)	2.26	(0.09)
Oregon	56.7	(53.3–60.0)	27.6	(24.6–30.8)	19.9	(17.3–22.8)	25.8	(22.9–28.9)	71.2	(68.0–74.1)	1.95	(0.07)
Pennsylvania	45.9	(42.3–49.5)	33.7	(30.5–37.2)	17.3	(14.6–20.3)	28.3	(25.1–31.7)	71.9	(68.6–74.9)	1.77	(0.07)
Rhode Island	48.8	(45.6–52.0)	30.0	(27.1–33.0)	17.5	(15.1–20.2)	27.9	(25.0–31.0)	71.5	(68.5–74.4)	1.77	(0.06)
Texas	54.7	(51.6–57.7)	28.6	(26.0–31.4)	19.1	(16.9–21.7)	32.1	(29.3–35.0)	73.3	(70.5–75.9)	1.92	(0.06)
Utah	50.1	(47.3–52.9)	26.5	(24.1–29.0)	14.8	(13.0–16.8)	22.7	(20.5–25.1)	67.2	(64.5–69.7)	1.54	(0.05)
Vermont	51.8	(48.8–54.9)	30.2	(27.4–33.1)	19.5	(17.1–22.1)	27.9	(25.2–30.7)	69.2	(66.4–71.9)	1.85	(0.06)
Washington	52.4	(49.1–55.7)	25.6	(22.8–28.7)	15.9	(13.6–18.6)	23.9	(21.2–26.8)	67.2	(64.0–70.2)	1.66	(0.06)
West Virginia	56.5	(53.2–59.6)	40.0	(36.9–43.2)	25.9	(23.1–28.8)	29.6	(26.7–32.6)	77.5	(74.7–80.1)	2.22	(0.07)
Wyoming	52.8	(49.1–56.4)	26.9	(23.8–30.2)	18.1	(15.5–21.1)	26.3	(23.2–29.6)	70.6	(67.2–73.8)	1.72	(0.06)
**Total**	**51.0**	**(50.1–51.9)**	**29.6**	**(28.8–30.4)**	**17.6**	**(16.9–18.3)**	**28.5**	**(27.7–29.4)**	**70.2**	**(69.3–71.0)**	**1.81**	**(0.02)**

**Abbreviations:** CI = confidence interval; SE = standard error.

* Alaska, Arkansas, Colorado, Delaware, Georgia, Hawaii, Maine, Maryland, Massachusetts, Michigan, Minnesota, Missouri, Nebraska, New Jersey, New York (excluding New York City), New York City, Ohio, Oklahoma, Oregon, Pennsylvania, Rhode Island, Texas, Utah, Vermont, Washington, West Virginia, and Wyoming.

† Percentages are weighted to reflect population-based estimates.

§ Excluding New York City.

**TABLE 2:** Self-reported prevalence of stressful life events (SLEs) in the year before an infant’s birth among mothers who had live births, by maternal demographic characteristics — Pregnancy Risk Assessment Monitoring System, 27 sites,* 2010

	≥1 financial SLE		≥1 emotional SLE		≥1 traumatic SLE		≥1 partner SLE		≥1 SLE total		Mean no of SLEs	
						
Characteristic	%†	(95% CI)	%†	(95% CI)	%†	(95% CI)	%†	(95% CI)	%†	(95% CI)	Mean	(SE)
**Age group (yrs)** §												
<25	62.2	(60.5–63.9)	33.2	(31.6–34.8)	27.4	(25.9–29.0)	40.6	(38.9–42.3)	80.0	(78.6–81.4)	2.43	(0.04)
25–29	51.6	(49.9–53.3)	28.4	(26.9–29.9)	15.7	(14.5–17.0)	24.7	(23.3–26.2)	69.5	(67.9–71.0)	1.72	(0.03)
≥30	41.8	(40.4–43.2)	27.7	(26.5–29.0)	11.2	(10.4–12.2)	21.8	(20.6–23.0)	63.0	(61.6–64.3)	1.38	(0.02)
**Race/Ethnicity** §												
White, non-Hispanic	48.2	(47.0–49.3)	30.9	(29.8–31.9)	16.0	(15.2–16.9)	25.1	(24.1–26.1)	68.5	(67.4–69.5)	1.70	(0.02)
Black, non-Hispanic	57.6	(55.3–60.0)	32.9	(30.8–35.0)	23.0	(21.1–25.0)	41.7	(39.5–44.0)	76.5	(74.3–78.5)	2.32	(0.06)
Hispanic	55.7	(53.2–58.2)	26.7	(24.6–29.0)	20.7	(18.7–22.8)	30.7	(28.4–33.1)	73.9	(71.7–76.0)	1.92	(0.05)
Asian/Pacific Islander	42.1	(38.9–45.4)	18.4	(16.0–21.0)	5.4	(4.3–6.8)	21.3	(18.6–24.2)	56.9	(53.5–60.2)	1.11	(0.04)
Other	58.7	(54.1–63.1)	30.7	(26.8–35.0)	21.3	(18.0–25.0)	31.6	(27.7–35.8)	73.3	(68.8–77.4)	2.04	(0.10)
**Education (yrs)** §												
≤12	57.5	(56.0–59.0)	30.0	(28.7–31.4)	24.2	(22.9–25.5)	34.9	(33.5–36.4)	75.6	(74.3–76.9)	2.16	(0.03)
13–15	56.0	(54.2–57.7)	32.2	(30.6–33.9)	18.5	(17.2–19.9)	31.5	(29.9–33.1)	73.7	(72.1–75.2)	2.01	(0.03)
≥16	37.4	(35.9–39.0)	26.8	(25.5–28.2)	7.5	(6.7–8.4)	16.8	(15.7–18.0)	59.6	(58.0–61.1)	1.13	(0.02)
**Marital status** §												
Married	44.4	(43.3–45.5)	28.0	(27.0–29.0)	10.9	(10.1–11.6)	19.5	(18.6–20.4)	64.2	(63.1–65.2)	1.38	(0.02)
Not married	61.3	(59.7–62.8)	32.1	(30.7–33.6)	28.1	(26.8–29.5)	42.6	(41.1–44.2)	79.6	(78.3–80.9)	2.48	(0.04)
**Health care coverage** §												
Medicaid	63.1	(61.6–64.6)	31.2	(29.8–32.6)	25.9	(24.6–27.2)	38.1	(36.6–39.6)	78.7	(77.5–80.0)	2.41	(0.03)
Not Medicaid	42.4	(41.3–43.6)	28.5	(27.4–29.5)	11.7	(10.9–12.5)	21.7	(20.8–22.7)	64.2	(63.1–65.3)	1.38	(0.02)

**Abbreviation:** CI = confidence interval.

* Alaska, Arkansas, Colorado, Delaware, Georgia, Hawaii, Maine, Maryland, Massachusetts, Michigan, Minnesota, Missouri, Nebraska, New Jersey, New York (excluding New York City), New York City, Ohio, Oklahoma, Oregon, Pennsylvania, Rhode Island, Texas, Utah, Vermont, Washington, West Virginia, and Wyoming.

† Percentages are weighted to reflect population-based estimates.

§ Chi-square value p≤0.05 for relationship of selected maternal demographic with prevalence of selected SLE type. P≤0.05 for difference in mean by analysis of variance.

## Footnotes

* Alaska, Arkansas, Colorado, Hawaii, Maine, Nebraska, Oklahoma, Utah, Washington, and West Virginia.

† Alaska, Arkansas, Colorado, Delaware, Georgia, Hawaii, Maine, Maryland, Massachusetts, Michigan, Minnesota, Missouri, Nebraska, New Jersey, New York (excluding New York City), New York City, Ohio, Oklahoma, Oregon, Pennsylvania, Rhode Island, Texas, Utah, Vermont, Washington, West Virginia, and Wyoming.
